# Participation in Advance Care Planning Among Medically At-Risk Rural Veterans: Protocol for a Personalized Engagement Model

**DOI:** 10.2196/55080

**Published:** 2024-04-12

**Authors:** Tammy Walkner, Daniel W Karr, Sarah Murray, Amanda Heeren, Maresi Berry-Stoelzle

**Affiliations:** 1 Veterans Rural Health Resource Center Iowa City VA Health Care System Iowa City, IA United States; 2 Center for Access & Delivery Research and Evaluation (CADRE) Iowa City VA Health Care System Iowa City, IA United States; 3 Iowa City VA Health Care System Iowa City, IA United States; 4 Department of Psychiatry Carver College of Medicine Unviersity of Iowa Iowa City, IA United States; 5 Vaughan Institute Tippie College of Business University of Iowa Iowa City, IA United States

**Keywords:** advance care planning, chronic disease, end-of-life care, health care decision, medical decision-making, recruiting, shared medical decision-making

## Abstract

**Background:**

Many of the challenges in advanced care planning (ACP) conversations are linked to the waxing and waning progress of serious illnesses. Conversations with patients about future medical care decisions by a surrogate decision maker have historically been left until late in the patient’s disease trajectory. These conversations often happen at a time when the patient is already very ill. The challenge in effective early ACP and serious illness conversations is to create a situation where patients appreciate the link between current and future medical care. Setting the stage to make these conversations more accessible includes using telehealth to have conversations at the patient’s place of choice. The personalization used includes addressing the current medical and social needs of the patient and ensuring that expressed needs are addressed as much as possible. Engaging patients in these conversations allows the documentation of patient preferences in the electronic health record (EHR), providing guidelines for future medical care.

**Objective:**

The objective of our telehealth serious illness care conversations program was to successfully recruit patients who lacked up-to-date documentation of ACP in their EHR. Once these patients were identified, we engaged in meaningful, structured conversations to address the veterans’ current needs and concerns. We developed a recruitment protocol that increased the uptake of rural veterans’ participation in serious illness care conversations and subsequent EHR documentation.

**Methods:**

The recruitment protocol outlined herein used administrative data to determine those patients who have not completed or updated formal ACP documentation in the EHR and who are at above-average risk for death in the next 3-5 years. The key features of the telehealth serious illness care conversations recruitment protocol involve tailoring the recruitment approach to address current patient concerns while emphasizing future medical decision-making.

**Results:**

As of September 2022, 196 veterans had completed this intervention. The recruitment method ensures that the timing of the intervention is patient driven, allowing for veterans to engage in ACP at a time and place convenient for them and their identified support persons.

**Conclusions:**

The recruitment protocol has been successful in actively involving patients in ACP conversations, leading to an uptick in completed formal documentation of ACP preferences within the EHR for this specific population. This documentation is then available to the medical team to guide future medical care.

**International Registered Report Identifier (IRRID):**

RR1-10.2196/55080

## Introduction

### Overview

Advance care planning (ACP) involves dialogues between patients and their health care providers regarding the patient’s values and health care preferences [1-3]. These discussions provide a foundation for health care providers and surrogate decision makers to make informed decisions in situations where the individual is unable to do so. Veterans Health Administration (VHA) offers ACP as a named health care benefit for enrolled veterans [4]. Advance directives (ADs) are documents outlining instructions for future medical care and go into effect only if an individual is not capable of making medical decisions or communicating their wishes [3]. Not all ACP sessions result in the completion of an AD, nor is completing an AD always the goal of specific ACP discussions [1,2]. This project focuses on increasing ACP participation and documentation among VHA enrolled veterans.

ACP is a key factor in end-of-life care, yet it is an underused intervention [5]. Among US veterans, it is estimated that 7.1 million of the 9.6 million veterans who are enrolled in VHA do not have ACP preferences documented in their medical records [1,6,7]. Work on AD discussions by Matthieu and colleagues [1] shows only 5.2% of all VHA beneficiaries had a documented ACP discussion in 2020.

Research suggests older adults may not be informed about the ACP process and may assume that physicians initiate conversations about advanced illness or end-of-life care [5,8]. Additionally, some older adults might lack adequate health literacy to understand the contents of end-of-life care conversations or the risks related to treatments they may receive throughout the illness trajectory [9-12]. Communication about end-of-life preferences may also be difficult if the health care provider or the patient is reluctant to initiate discussion or the provider lacks sufficient time to engage in the conversation [1,9,13,14]. Yet there are benefits to ACP, such as reducing uncertainty when a patient is unable to communicate their wishes, receiving care that is more congruent with personal preferences, and including care that is more consistent with the spiritual cultural needs of patients and their families [14-17]. Additionally, for specific high-risk populations, the presence of an advanced care plan before hospital admission has been shown to decrease intensive care unit length of stay [8,18,19].

VHA supports multiple approaches to facilitate ACP conversations between patients, surrogate decision makers, and providers, including, but not limited to, the serious illness care conversation (SICC), the goals of care conversations, and the Life-Sustaining Treatment Decisions Initiative [4,20]. The National Center for Ethics in Health Care is at the forefront of making ACP information available to veterans and the Veterans Administration’s (VA) clinical and nonclinical workforce. National Center for Ethics in Health Care has been central to providing guidelines and training to support ACP practices within VHA. Enterprise-wide initiatives, such as advance care planning through group visits, are additional offerings at various VA sites that are successfully increasing access to ACP [1,21]. VHA has been promoting life-sustaining treatment (LST) as the AD of choice for VHA; however, the limitation of LSTs to VA health systems restricts the transferability of the veteran’s wishes to community-based hospitals and providers. LSTs are ADs that are only valid in VA health facilities and are not widely accepted outside of VHA. Despite known benefits and resources for providers and veterans for engaging in ACP, documentation of ACP discussions in veterans’ electronic health records (EHRs) remains low [1].

### Challenges of ACP in a Rural Veteran Population

Rural veterans living with serious illness, compared to their urban peers, have increased barriers to ACP within VHA and community health care systems [15,22,23]. Serious illness has been defined as a “health condition that carries a high risk of mortality and either negatively impacts a person’s daily function or quality of life or excessively strains their caregivers” [24]. Veterans who rely on VHA for care are often sicker, tend to live in rural areas, and face significant travel challenges regarding broad geographic distance and seasonal weather impacting travel safety [22,23]. While the VA Mission Act of 2018 has increased access to medical care for rural veterans [25], formalizing ACP processes still lags for rural veterans [1,26]. Access to medical care is a concern for many rural veterans, and with fewer care options, there are fewer opportunities to talk about serious illness and advance care planning [15,27]. Urban-dwelling veterans often face similar challenges, so any improvements to access through these interventions have the potential to benefit all veterans.

Many rural veterans use both VA and community-based health care [28,29], indicating a need for ACP that is translatable to both VA and community-obtained health care. As such, this intervention seeks to promote ACP and offers support to veterans wishing to complete whichever formal AD documentation format is best suited for their unique health care needs. For veterans, whose primary care location is VA, LST offers a nuanced format for documenting future care decisions. For those, whose primary site of care is outside VA, the state-based AD, such as the durable power of attorney form, should be filled out.

SICC acknowledge that the disease is unlikely to resolve, and managing symptoms and medical crises is an ongoing challenge for patients and their support team [2-4,30]. While SICCs are widely applicable, they are an underutilized structure for conversations between a provider and a patient who is focusing on living with a serious illness [9,31]. The telehealth serious illness care conversation (tSICC) adapts many of the tenets of SICC for a telehealth intervention while utilizing VA clinical standards designed for ACP [32]. These frameworks have been adjusted to suit conversations with veterans in their homes who are not expected to be in a current medical crisis [4,32]. The provider talks with the patient about their values and priorities for health care, as well as how those might change in the context of advancing illness with or without an acute medical crisis. The goal is more than simply discussing resuscitation status; but rather, it is to develop a personalized plan for medical care as the patient’s illness progresses. While SICC acts as an entry into the ACP process, these types of conversations often happen too late in a person’s medical trajectory [16,31]. Early engagement allows for relational decision-making for veterans and their families [33], accommodating the more complex information about the variable clinical trajectory of older and more seriously ill veterans.

Personalized approaches have been shown to promote engagement in the ACP process [5,34]. Our project tailored the recruitment strategies for rural veterans, recognizing they have more barriers to receiving end-of-life health care, including limited access to hospice and palliative care services [15]. This manuscript outlines a recruitment protocol for rural, seriously ill veterans into a quality improvement (QI) project at a veteran healthcare system in the Midwestern United States.

To optimize integration of veteran-specific values, this QI project used community-based participatory research (CBPR) practices [35-37]. According to National Institutes of Health, CBPR involves researchers and community members to combine “knowledge with action to improve health outcomes and eliminate health disparities” [38]. To accomplish our goal of developing a veteran-centric approach to engaging in ACP, we established a team that included veterans and veterans’ proxy decision makers (through a Veteran Engagement Panel [VEP]), an advocacy expert, social workers, licensed VA medical providers, and veteran service officers. The VEP was convened specifically to provide guidance for the pilot, accompanying the project from institutional review board (IRB) approval through the development of the intervention and analysis of preliminary results. The VEP was integral to the development of veteran-facing materials, including flyers and other recruitment materials, as well as the development of metrics reflecting veteran priorities. Panel members shared their insights on how to personalize all aspects of the study to support the goal of making the process relatable to the veteran population.

### A Telehealth-Based, Multimodal, and Veteran-Centric Intervention

Improving access and decreasing barriers were driving forces in our QI project: develop and disseminate a veteran-centric clinical protocol for ACP through tSICC. Telehealth visits expand access for patients who face barriers to receiving medical care [39-41]. However, technology issues limit the use of telehealth as an equitable or appropriate care mechanism [39]. While VHA has been using telehealth for many years, rural veterans’ limited access to high-quality broadband service can be a barrier to telehealth services [42,43]. Evidence has suggested veterans with a serious illness were satisfied with telehealth encounters, as these types of appointments improved their access to care and diminished the need for travel [39].

### Information About the tSICC Pilot

Our project targets at-risk veterans with no ADs or out-of-date AD documentation in veterans’ EHRs. Our team obtained funding through VHA Office of Rural Health for a QI project within VHA; we are a rural-serving Midwest VA medical center. We present here a systematic description of a stakeholder informed, multimodal recruitment strategy for a clinical intervention for ACP.

### Setting for the Development of the Engagement Strategy

Our QI project sought to determine the appropriate timing and setting for a tSICC within a veteran’s clinical trajectory. We had a driving slogan: “the right care, for the right veteran, at the right time.” The steps chosen for recruitment were consistent with local IRB guidelines, local and national VA practices, and were influenced by veteran feedback [32]. To our knowledge, there is no specific literature outlining the development of a veteran-centric recruitment model for telehealth-based ACP.

### Objective

This manuscript describes a recruitment protocol for engaging veterans who have not been reached through existing opportunities to participate in a tSICC and ACP within VHA.

## Methods

### Study Context

This paper reports on the methodology of an initial pilot study that is part of a larger clinical work-flow QI project to increase veteran engagement in and documentation of ACP within VHA. Our team is developing a telehealth-based approach to an existing clinical intervention, the SICC, with a focus on allowing the veteran and their chosen support persons to participate in ACP on their own timeline and in a setting that is convenient and comfortable for them. This is especially important for rural veterans who may face a significant travel burden to access care at VHA. Veterans and their families often accommodate this burden by cramming many of their medical appointments into one day. However, reducing the travel burden can lead to a busy, medically-focused schedule and increased fatigue. The lack of time during clinical visits is a barrier for interventions like SICC that promote ACP [31,39,44]. By removing this clinical intervention from an already taxing day and creating a space for this important conversation to occur outside of stressful clinical visit days, we hypothesized that veterans would be better able to participate in ACP. Consequently, as this discussion is documented in the EHR and veterans are guided on how to have future ACP conversations, there is a prospect for increased efficacy in future discussions of ACP among veteran participants and their VHA medical providers and social workers.

Engaging participants in research or QI studies can be difficult, especially if participants are seriously ill or the study is focused on a sensitive issue such as ACP. Further study of clinical interventions can help identify better strategies for recruitment and participation in research focused on ACP and palliative care [44,45]. Studies have shown that using multiple strategies in the recruiting phase may help increase participation related to ACP [46-48]. Chau and colleagues [46] demonstrated that mailing information along with making phone calls to participants is an effective method for increasing their willingness to engage in the ACP process. For this QI project, an assortment of methods were used to recruit participants to the ACP process.

### Study Design

The tSICC intervention was implemented in a medium-sized VA health care system in a Midwestern city in the central United States with a regional metropolitan population of 171,000. VHA EHR system was used for population-based identification of medically at-risk veterans. The pilot cohort was recruited from July 2021 to October 2021 using a project-developed risk metric that identifies and categorizes by risk level those who might experience a serious health episode in the next 5 years. Veterans were identified as not having an AD or other VHA-based ACP documentation in their EHR within the last 2 years. Veterans were receiving care through VHA but may also be supplementing it with community-based care. The recruitment goal was for 200 veterans to participate in the intervention.

While the intervention was planned as a telehealth intervention, recruitment was initially done during a clinical appointment starting in April 2021. The project team quickly identified that in-clinic recruitment was difficult due to existing workflows that did not easily integrate the recruitment process. This experience is noted in the literature, with previous works [33,40] showing that having ACP conversations as part of the clinical workflow is challenging and does not engage populations that may have significant comorbidities and more pressing medical concerns. In October 2021, the study team transitioned to an entirely remote, multimodal recruitment process.

Applying CBPR principles to further enhance veteran-centricity, veteran participants were invited to take part in a concise survey of their experience in both the recruitment process and the intervention. Evaluation questions from the CollaboRATE survey, based on core elements of shared decision-making [49], were used in a postintervention call to assess if participants felt informed about the purpose of the intervention and if they felt they were part of the decision-making process [50]. The collaboRATE questions have been delivered through multiple modalities (eg, paper, digital tablet, and social media) [50,51], which makes this short survey a viable option for individualized projects such as our QI pilot. Feedback from these questions was an important element in adapting the pilot study to veteran-identified concerns and wishes.

### Study Protocol

**Figure 1:**
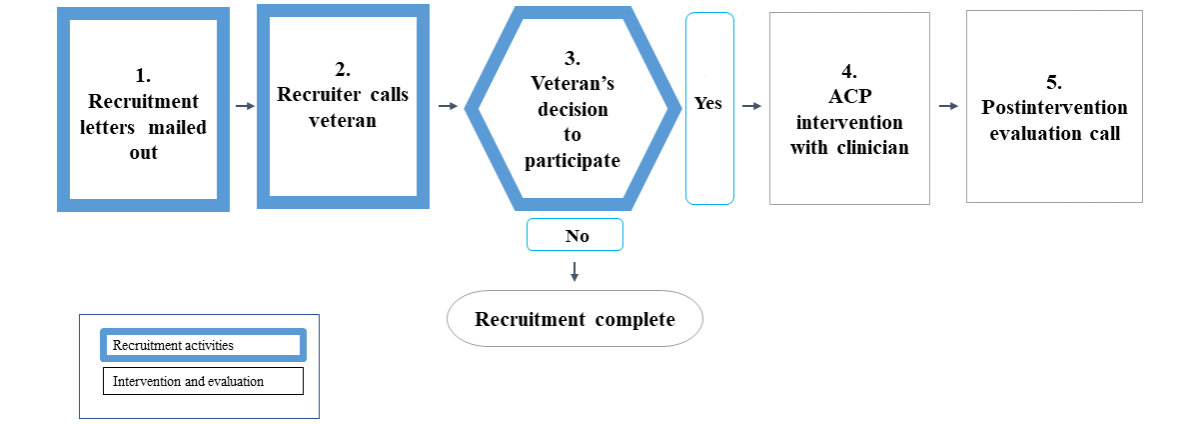
Overview of the quality improvement recruitment process. ACP: advanced care planning.

**Figure 2:**
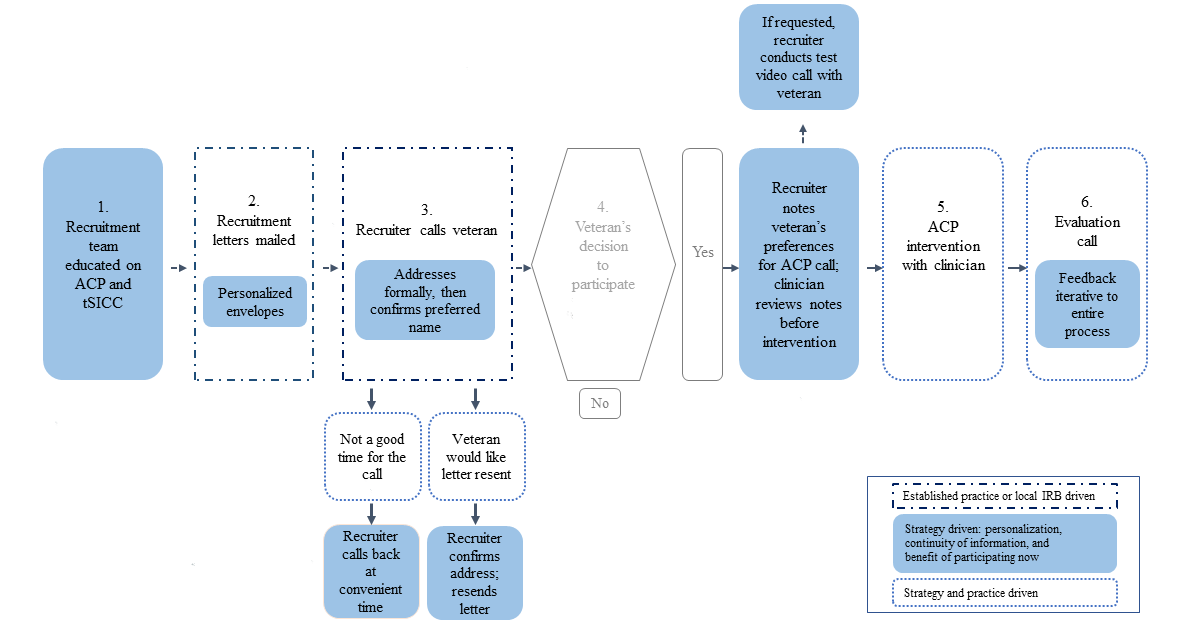
A detailed view of the personalized recruiting process. ACP: advanced care planning; IRB: institutional review board; tSICC: telehealth serious illness care conversations.

The required steps of local IRB and VA offices at the time of this intervention development were mail notification before placing up to 3 unsolicited phone calls and leaving up to 1 unsolicited voicemail. The parameter aligns with evidence that 3 phone calls promote engagement and minimize fatigue [34]. In addition to institutional guidelines, the recruitment strategy was shaped by our work with the VEP, which identified 3 concepts that became guideposts for the recruitment strategy: personalization, continuity of information, and the benefit of participating in ACP now. See Figure 1 below for an overview of the major steps in our pilot project. Figure 2 offers a more detailed view of our process.

To personalize the mailing phase of recruitment, special attention was given to the items being sent to the veteran. Large manilla envelopes were hand-addressed in blue ink to appear unique among other pieces of mail and later be referenced by color and size during recruitment phone calls.

Several strategies were available during phone recruitment to personalize the experience for the veteran. The recruiter, trained on the components of ACP, reviewed the veteran’s medical record before the call to ensure familiarity with the veteran’s preferred name and correct address, identify any recent major medical events, and obtain the name of the veteran’s emergency contact or next of kin (inviting the veteran to include them in the tSICC if they wished). During the call, attention was paid to the veteran’s health literacy and comfort with technology. The recruiter estimated the veteran’s understanding of the project and if the veteran might need technical assistance (ie, in preparing for video chat) to participate in the telehealth intervention. When needed, further explanation was provided, or plans were made to provide technical assistance before the intervention.

Continuity of information can be challenging in any large health care system, and veterans may feel that information is not shared among members of the health care team. This was reported as a negative experience by the VEP and indicated decreased confidence in both competency and compassion from their care team. To address that concern, the recruitment team used a formal hand-off to ensure that concerns and topics raised by the veteran during the recruitment call were passed on to the next team member. During recruitment, veterans were informed that they would have an opportunity to talk with a VA medical provider about their general concerns related to ACP. Concerns shared during the recruitment phone call would be relayed to the provider and could be discussed further during the telehealth visit.

The final concept proffered by the VEP regarded the benefit of this ACP intervention and said that every interaction with the veteran should be of potential benefit. Early in the disease process, veterans are active decision makers, and tSICCs are grounded in patient autonomy. The tSICC protocol offers ACP at the veteran’s convenience rather than during a busy clinic schedule or a time of medical crisis, creating time and space for veterans to ask for help with medical decision-making. In line with the project’s objective to enhance veteran comprehension of ACP and the support available from VA, veterans were informed that the health care provider involved in this intervention could assist them in translating their values, goals, and wishes into future treatment decisions, all of which would be recorded in their VA EHR. This included how completing an AD can be a significant step in gaining agency in their future medical decision-making.

### How the Principle of Tailoring Follows Through Subsequent Steps

Veterans successfully recruited for the project participated in the tSICC intervention and a postintervention follow-up call. During the intervention, the VA provider was sure to ask about topics important to the veteran and their family that may have been identified during the recruiting phone call. The VA provider then documented the ACP conversation in the veteran’s EHR. As needed, appropriate referrals were made to VA providers, and relevant resources were shared with the veteran.

Following the intervention, veterans were invited to participate in the evaluation of the recruitment and intervention protocols. To assess the appropriateness, timeliness, and usefulness of the intervention, there was a 3-item questionnaire as well as a query of their opinion about the intervention and any suggestions they may have for improving the process. An iterative process was used to incorporate input from veterans that shaped continued recruitment procedures. The concluding phone call also provided a chance to address any inquiries regarding ACP or the tSICC and to ensure the completion of any previous tasks.

### Analysis of Project-Generated Engagement Data

Data collection on recruitment focused on monitoring the number of attempts, the results of these attempts, and veteran-reported satisfaction with the recruitment process. The structure of the phone call outcomes gave insight into the challenges of reaching veterans at home (Textbox 1).

**The possible outcomes for telephone recruiting.**

**Outcomes of phone calls**

- Message left
- Did not leave message
- Telephone problem
- Wrong number or disconnected
- Deceased
- Call back requested
- Declined
- Asked for more information
- Resend letter
- Agreed to participate
- Refused to participate

If the recruiter got a message “No longer in service,” “Busy signal,” or “Unable to leave a message,” it was not logged as first contact; those were simple telephone problems or technical issues. Some cellular services provide a message if the call cannot be completed as dialed. If the issue was telephone company-related and not customer-related, it was not considered a contact.

### Ethical Considerations

This project was determined as quality improvement nonhuman participants research by the University of Iowa IRB for January 2020 (#201911479), March 2020 (#202002148), and April 2021 (#202102175). We did not require a waiver or informed consent as this intervention was nonhuman subject research. All study data for this QI project has been deidentified, and no personal information has been reported in this paper. There was no compensation since this project was nonhuman subject research.

## Results

This project seeks to understand how VHA can deliver ACP that supports “the right care, for the right veteran, at the right time.” Here we report the results of the feedback received from veterans in the development of the recruitment process and its integration into our ongoing recruitment for this multiyear project. VEP and intervention participants provided the insight that 3 concepts should be at the forefront of our approach: personalization, continuity of information, and a benefit to the veteran now.

Personalization occurred at all levels of the recruitment protocol. Our team aimed to have mailed materials “stand out” against mail that could otherwise be perceived as junk by using larger envelopes and hand-addressing them. We also customized recruitment discussions (and subsequent intervention discussions) to align with the veteran’s ACP experiences to date. Further, project team members recognized and addressed the individual needs of the veteran as identified in the EHR and from previous interactions with the project team. By not repeating already shared information, veterans, and staff saved time. This allowed conversations to move to solutions for veterans’ concerns and building next steps for planning more efficiently.

Immediate benefits to veterans from the intervention included: provider documentation of veterans’ values and personal health goals to be used as guidance to future proxy decision makers; updating incorrect or incomplete next-of-kin information stored in the EHR; and assistance in the completion of AD to be entered into the EHR. Further, increasing a veteran’s understanding of ACP and how VHA can help the veteran with future medical decision-making was a vital component of the clinical intervention and, as such, was integrated into every contact, starting at recruitment. To facilitate this, our recruiter was knowledgeable of ACP principles and the tSICC intervention and had the necessary resources to explain the project and the background verbally in electronic and paper form.

The impact of the recruitment strategy was that of the 343 veterans successfully reached during recruitment by phone, 199 agreed to participate, and 196 completed the intervention. From this group, 146 completed the post call evaluation. Veteran satisfaction with the intervention is reported in Table 1. Notably, 50% (98/196) of veterans who completed the intervention then requested to have AD forms mailed to them for completion. When asked whether the timing of the tSICC was appropriate and timely for them, 66.4% (97/146) of respondents agreed it was.

**Table 1:** Veterans’ satisfaction with a telehealth Serious Illness Care Conversation intervention (N=146).

Veteran agreement with elements of satisfaction			Value, n (%)
**Provider understood patient’s values**			
	Rural^a^	86 (76.1)	
	Urban^a^	23 (65.7)	
	Total	109 (74.7)	
**Provider includes what matters most**			
	Rural^a^	83 (73.4)	
	Urban^a^	22 (66.7)	
	Total	105 (71.9)	
**Appropriate timing of conversation**			
	Rural^a^	75 (66.3)	
	Urban^a^	22 (66.7)	
	Total	97 (66.4)	

^a^Rural-Urban Commuting Area Codes guidelines were used to determine rural or urban status [52].

The intervention and related data collection for the ongoing QI project are expected to be completed in the fiscal year 2024. The results of that data are forthcoming.

## Discussion

The results of this QI project support the hypothesis that veterans are more likely to engage in ACP if the conversation is timely, not perceived as burdensome, and addresses veterans’ current medical and related concerns [5,14,41]. The recruitment method outlined herein offers personalized strategies to engage veterans in ACP outside of a clinical setting that were informed and evaluated by veterans.

The QI team adapted VA trainings on advance care and serious illness conversations to be used in a short, home telehealth setting and focused on identifying a process to engage veterans in a way that fits their unique needs. The digital divide was identified as a significant barrier for rural veterans [42-44]. Our findings are consistent with previous results, which show that offering lower tech communication options as well as support for connectivity in the form of personalized assistance lowered systemic barriers to access [43,46].

Data reported in the literature and gathered from our dedicated VEP underscores the importance of the veteran’s perception that their unique needs and perspectives are important to the ACP process [39]. This both serves to build rapport and to tailor subsequent information offered in the course of the intervention about ACP. Particular focus was on how engaging in ACP now can help inform their current medical decision-making as well as the benefit of formal documentation of ACP within the veteran’s EHR [9,14].

A notable limitation of this study is that while we documented increases in ACP behaviors across the sample, we did not capture uniform, preintervention data that may have provided insight into the unique barriers and facilitators that influence an individual’s engagement in ACP. Another limitation is the study’s small size, at 1 Midwest VA medical center. This important limitation will require further study before any generalization, ideally as a multisite pilot.

The recruitment strategies used over the course of this QI project support veterans in accessing and understanding ACP by addressing both common and unique barriers to engaging in ACP. Furthermore, this project demonstrates that telehealth is a viable mechanism to provide meaningful and satisfactory recruitment of veterans into the ACP process. Overall, we show that personalization in recruitment allows veterans to see that their needs are being met by VHA services. Demonstrating sensitivity to current veteran needs can increase access to the delivery of ACP. This approach is clinically feasible as a telehealth intervention. We demonstrate that a telehealth protocol with personalization and continuity of communication increases the engagement of veterans in clinical interventions about sensitive issues such as ACP and SICC. Engaging veterans in these conversations and documenting the results in the EHR is the first important step in ensuring veteran-centric care in times of medical crisis.

## Abbreviations

ACP: advanced care planning
AD: advance directive
CBPR: community-based participatory research
EHR: electronic health record
IRB: institutional review board
LST: life-sustaining treatment
QI: quality improvement
SICC: serious illness care conversations
tSICC: telehealth serious illness care conversations
VA: Veterans Administration
VEP: Veteran Engagement Panel
VHA: Veterans Health Administration
